# COMT val158met Polymorphism and Neural Pain Processing

**DOI:** 10.1371/journal.pone.0023658

**Published:** 2012-01-11

**Authors:** Christian Schmahl, Petra Ludäscher, Wolfgang Greffrath, Anja Kraus, Gabriele Valerius, Thomas G. Schulze, Jens Treutlein, Marcella Rietschel, Michael N. Smolka, Martin Bohus

**Affiliations:** 1 Department of Psychosomatic Medicine and Psychotherapy, Central Institute of Mental Health, Mannheim, Germany; 2 Division of Neurophysiology, Center of Biomedicine and Medical Technology, Mannheim, Germany; 3 Department of Psychiatry and Psychotherapy, University Medical Center, Goettingen, Germany; 4 Department of Genetic Epidemiology in Psychiatry, Central Institute of Mental Health, Mannheim, Germany; 5 Department of Psychiatry and Psychotherapy, Technische Universität Dresden, Dresden, Germany; The University of Melbourne, Australia

## Abstract

A functional polymorphism (*val158met*) of the gene coding for Catechol-O-methyltransferase (COM) has been demonstrated to be related to processing of emotional stimuli. Also, this polymorphism has been found to be associated with pain regulation in healthy subjects. Therefore, we investigated a possible influence of this polymorphism on pain processing in healthy persons as well as in subjects with markedly reduced pain sensitivity in the context of Borderline Personality Disorder (BPD). Fifty females (25 patients with BPD and 25 healthy control participants) were included in this study. Genotype had a significant – though moderate - effect on pain sensitivity, but only in healthies. The number of *val* alleles was correlated with the BOLD response in several pain-processing brain regions, including dorsolateral prefrontal cortex, posterior parietal cortex, lateral globus pallidus, anterior and posterior insula. Within the subgroup of healthy participants, the number of *val* alleles was positively correlated with the BOLD response in posterior parietal, posterior cingulate, and dorsolateral prefrontal cortex. BPD patients revealed a positive correlation between the number of *val* alleles and BOLD signal in anterior and posterior insula. Thus, our data show that the *val158met* polymorphism in the COMT gene contributes significantly to inter-individual differences in neural pain processing: in healthy people, this polymorphism was more related to cognitive aspects of pain processing, whereas BPD patients with reduced pain sensitivity showed an association with activity in brain regions related to affective pain processing.

## Introduction

Neural processing of pain has different components besides somatosensory processing [1], [2], with affective aspects being processed in anterior insula, anterior cingulate cortex (ACC) and amygdala and cognitive aspects in posterior parietal and dorsolateral prefrontal cortex (DLPFC). DLPFC participates in a pain control network, which regulates cortico-subcortical and cortico-cortical loops by inhibiting ascending pathways through the anterior cingulate cortex [3].

Besides its role in cognitive function, particularly in the prefrontal cortex [4], genetic variations in the gene coding for the enzyme Catechol-O-methyltransferase (COMT) are associated with the processing of emotional stimuli in limbic and prefrontal regions [5], [6]. A common functional genetic variation in the COMT gene is the *val158met* polymorphism with the *val* variant showing higher enzyme activity than the *met* variant [7].

In relation to pain, decreased dopamine neurotransmission, as conferred by increased COMT activity, is related to increased enkephaline levels and, in turn, a down-regulation of μ-opioid receptors [8]. Variations in the gene coding for COMT explain approximately 10% of variability in pain sensitivity [9]. The influence of the *val158met* polymorphism appears to be particularly related to the temporal summation of longer lasting pain stimuli [4]. This polymorphism has been associated with conditions of increased pain [9]–[11]. *Met/met* individuals also showed higher pain levels following a single opioid dose [12]. Increased enzyme activity was associated with higher capacity to activate μ-opioid neurotransmission in thalamus, basal ganglia, limbic and paralimbic areas in response to a sustained pain challenge resulting in decreased pain perception [8].

A psychiatric condition characterized by reduced pain sensitivity is Borderline Personality Disorder (BPD) and the endogenous opioid system appears to play an important role in this domain [13]–[15]. According to the most recent study by Distel et al. [16], genetic aspects explain 35 to 45% of the variance in BPD. Patients suffering from BPD frequently experience stress-induced states with reduced pain perception, which are often relieved by self-injurious behavior [17]. Reduced pain sensitivity in patients with BPD was found under non-stress as well as under stress conditions [18]–[20]. BPD patients also exhibited greater levels of brain activation in dorsolateral prefrontal cortex and neural deactivation in perigenual anterior cingulate gyrus and amygdala suggesting a modulation of pain circuits, primarily through the downregulation of the affective components of pain [21], [22].

The aim of the current study was to investigate the influence of the *val158met* polymorphism on neural pain processing. Therefore we investigated healthy female subjects as well as patients with BPD to obtain a large variance in pain sensitivity. Overall we expected the number of *val* alleles to be correlated with brain activity in pain-processing brain regions. Given the above mentioned differences between both groups in affective and cognitive pain-evaluation, we expected different activation patterns in brain areas such as anterior insula, anterior cingulate cortex, and DLPFC.

## Methods

### Ethics Statement

The study was conducted in accordance with the declaration of Helsinki and was approved by the Ethics Committee of the Medical Faculty of Mannheim, Heidelberg University. After full explanation of the study, written informed consent was obtained from all participants.

### Subjects

Twenty- five healthy controls [age: 27.9±7.9] and twenty-five female patients diagnosed with BPD [age: 27.5±7.1] according to DSM-IV-criteria and matched according to age participated in this study. Pain processing data of 22 patients and 12 controls have been published previously [21]. All patients were either inpatients at the Dept. of Psychosomatic Medicine or were recruited via announcement on different BPD-specific homepages. Axis I and II diagnoses were assessed in all participants by trained psychologists using the Structured Clinical Interview for Axis I disorders (SCID) [23] and the International Personality Disorder Examination (IPDE) [24], respectively. The inter-rater reliability for the IPDE was κ = .77. All participants were free of psychotropic medication for at least two weeks prior to study. All subjects were right-handed as measured by the Edinburgh handedness inventory [25]. Exclusion criteria comprised neurological diseases, retained metal, history of head trauma, current depression, alcohol or substance abuse or dependence, lifetime bipolar I disorder, schizophrenia and pain disorders. Healthy controls were free of any Axis I or II disorder. After full explanation of the study, written informed consent was obtained.

### Neuroimaging

The methods used to characterize neural pain processing by functional magnetic resonance imaging (fMRI) have been described elsewhere [21], [22]. Briefly, heat stimuli were applied on the back of the right hand using a peltier based thermode (3×3 cm) controlled by a PC-based device (Thermal Sensory Analyzer, Medoc, Israel). Subjective pain intensity was rated by the participants after each stimulus using a numerical rating scale (NRS) ranging from 0 to 100 (“no pain” to “most intense pain imaginable”). All participants were measured on a 1.5 T scanner equipped with a Vision gradient system and a CP head coil (Siemens Medical Solution, Erlangen, Germany). For whole-brain structural volumes, we used a T1-weighted three-dimensional magnetization-prepared rapid acquisition gradient echo sequence with a voxel size of 1 mm^3^ in all subjects. The blood oxygen level-dependent (BOLD) signal was acquired using gradient-recalled echo-planar imaging (number of contiguous transversal slices, 25; position, covering all but the apical 20 mm of the brain; slice thickness, 5 mm; echo time, 60 ms; flip angle, 60°; matrix, 64×64 pixels; volume repetition time, 4175 ms, field of view 220×220 mm^2^, volume acquisition times 2600 ms, silent periods between scans 1575 ms). The silent periods interspersed between scans were used to ask the subjects for pain intensity evaluation after the first post-stimulation scan and to obtain their rating after the subsequent scan. During the acquisition of the functional data, ten stimulation blocks lasting 30 s each were applied. Five blocks consisted of a fixed moderate intense stimulus temperature (43°C, *condition 1*) and five blocks consisted of a higher intense stimulus temperature individually adjusted to correspond with a subjective pain intensity of 40% (*condition 2*) as determined in a psychophysical experiment directly before the scanning session (cf. Schmahl et al 2006, [22]). All given stimulus temperatures oscillated with an amplitude of 2°C to avoid adaptation [21], [22]. Stimulation blocks were interrupted by 60 s intervals with neutral temperature (35°C).

Data were analyzed with Statistical Parametric Mapping (SPM5, Wellcome Trust Centre for Neuroimaging, University College London, London, UK). Following pre-processing (spatial realignment, normalisation to MNI EPI template, smoothing: kernel width: 8×8×8 mm), the two conditions (painful [individual or fixed] vs. neutral temperature) were modelled as explanatory variables within the context of the general linear model. To detect associations between COMT genotype and fMRI activation, the contrast images (positive (1) and negative (2) association between COMT genotype and fMRI activation) of all subjects were included in a second level regression analysis (statistical threshold: p≤0.001, uncorrected, k>10). To model the assumed gene–dose effect, COMT genotype was coded as a covariate by the number of *val* alleles (0, 1, or 2) In addition, we used SPSS for Windows 15.0 to conduct non-parametric Spearman-Rho correlation analyses (“ROI analysis”) between the number of *val* alleles and the mean regression coefficients (betas) per voxel for each ROI (which showed significant correlations between BOLD signal and the number of *val* alleles in the regression analysis in one of the two groups). To compare healthy controls and patients with BPD regarding pain sensitivity on low and high pain stimuli, we conducted an ANOVA with post-hoc Scheffé analyses.

### Genotyping

Leucocyte genomic DNA from the 50 participants was isolated from EDTA anti-coagulated venous blood samples with the QIAamp DNA extraction kit (Qiagen, Chatsworth, CA, USA). Genotyping of the single nucleotide polymorphism (SNP) rs4680, was performed using TaqMan®, Drug Metabolism Genotyping Assays (Assay ID C_25746809_50) and protocol (7900HT Fast Real-Time-PCR-System, Applied Biosystems, Foster City, CA, USA). For quality reasons 15% of SNPs were typed twice and genotype replicate consistency was 100%. Hardy-Weinberg equilibrium (HWE) was assessed using the exact text implemented in the DeFinetti program (Strom, T.M. & Wienker, T.F. DeFinetti program at http://ihg2.helmholtzmuenchen.de/cgi-bin/hw/hwa1.pl).

## Results

### Genotype distribution

In the total group of 50 participants, 12 were homozygous for the *met158* allele of the COMT polymorphism, 26 were heterozygous, and 12 were homozygous for *val158*. The genotype distribution of the controls did not deviate from Hardy-Weinberg equilibrium (p = 1.00).

**Figure 1:**
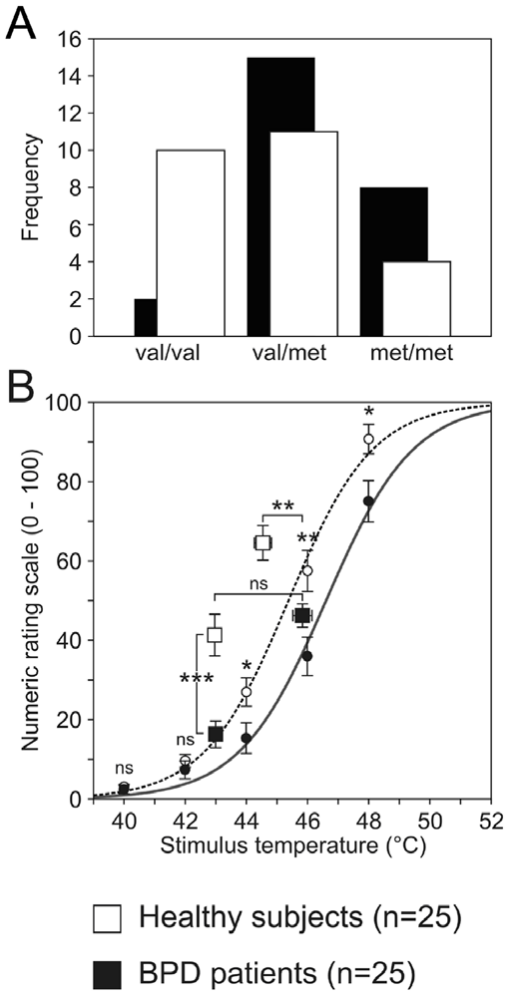
Patients suffering from BPD are less heat pain sensitive independent of the *val158met* polymorphism. (A) Genotype distribution of the *val158met* polymorphism in healthy controls (open) and BPD patients (filled bars) do not differ. (B) Heat pain perception was markedly reduced in BPD. Dose-response function in the pretest session (circles) and stimulus temperatures applied during fMRI (squares) did significantly differ between controls and BPD (ns p>0.4; * p<0.05; ** p<0.01; *** p<0.001, students t-test).

Eight patients in the BPD group were homozygous for *met158*, 15 were heterozygous and two were homozygous for *val158*. Of the healthy controls, four subjects were homozygous for the *met* allele, eleven were heterozygous and ten homozygous for *val* (Figure 1A).

### Subjective heat pain sensations

In proper replication of our previous studies [20], [22], [26], subjective heat pain perception was significantly lower in patients as compared to the control group. (Figure 1B). The mean stimulus temperatures to induce an equal subjective painful sensation was more than 1°C lower in BPD as compared to healthy controls (p<0.005). Therefore, during the fMRI-scanning procedure the lower stimulation with 43°C (condition 1) induced comparable subjective heat pain sensations in healthy controls as the higher heat stimuli were applied in BPD in *condition 2* (Figure 1B).

### Influence of COMT val158met on pain perception

**Figure 2:**
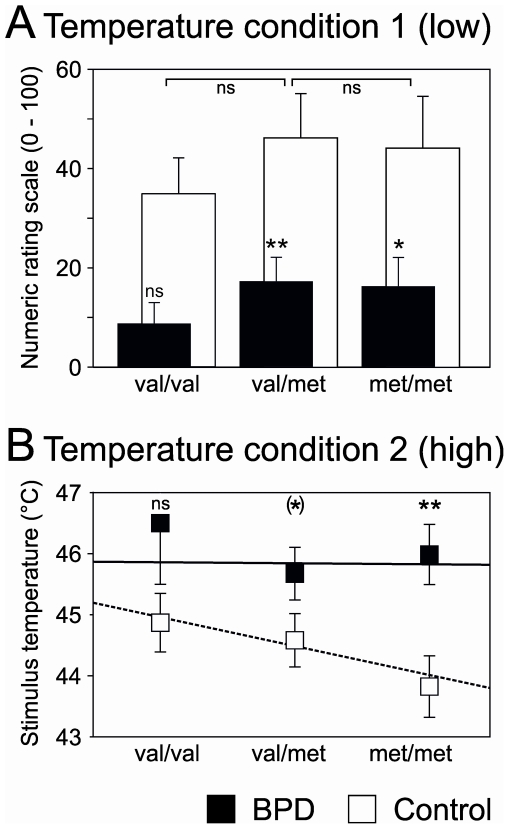
Association between COMT genotype and pain sensitivity. A: Lower heat pain perception in BPD in response to 43°C does not depend upon the *val158met* polymorphism. ^ns^ p>0.3, BPD and control, respectively; * p<0.05; ** p<0.01, students t-test. B: Stimulus temperatures for the induction of a heat pain sensation of NRS 40 decreases with the number of met alleles in healthy volunteers but not in BPD patients (ns p>0.19; (*) p<0.1; * p<0.05, students t-test).

When comparing the heat pain obtained in subjects with respect to the different genotypes, the equal heat stimuli of lower intensity given in *condition 1* still gave rise to significantly lower ratings in BPD irrespective of the *COMT val158met* polymorphism. Differences in genotype had no influence on heat pain perception neither in healthy controls nor in BPD patients. (Fig. 2A; ANOVA: main effect group: F = 11,9, df = 1, p<0.01; main effect genotype: F = 0,51, df = 2, p = 0.61; interaction effect: F = 0,01, df = 2, p = 0.99). Interestingly however, the mean stimulus temperatures for a subjective pain rating of 40 displayed nominally an inverse relation with genotype in the healthy participants (Fig. 2B; healthy r = 0.97, p = 0.16; BPD r = 0.61; p = 0.58).

### Influence of COMT val158met on neural pain processing

**Figure 3:**
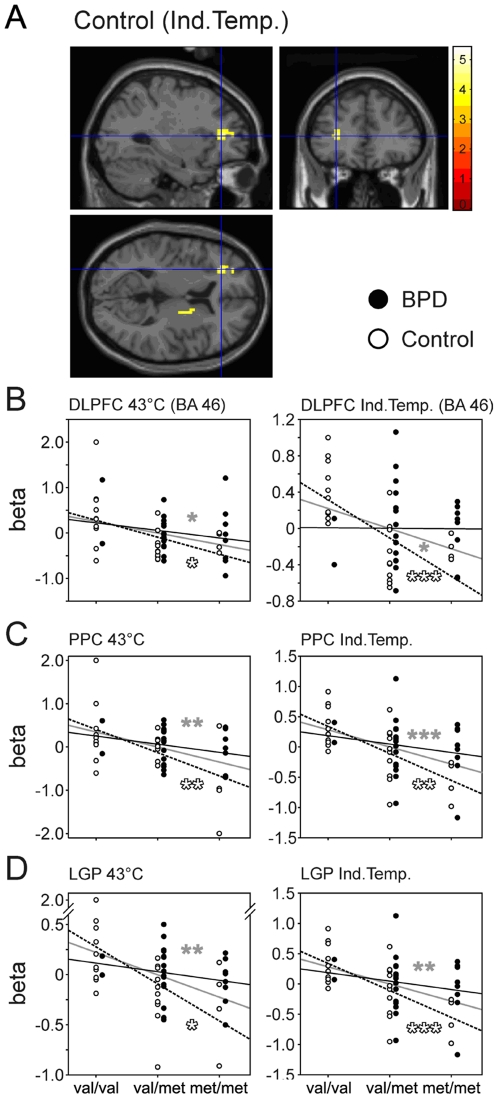
Association between COMT genotype and fMRI activation during painful stimulation in frontal brain areas. Significant correlations were observed for both stimulus conditions within the dorsolateral prefrontal cortex (DLPFC; A,B). Furthermore, the posterior parietal cortex (PPC; C) and the lateral globus pallidus (LGP; D) also displayed significant correlation during both conditions. Statistics and regression lines for group means are shown in gray (all 50 subjects), black (BPD), and dashed (Control), respectively; significant Pearson product moment correlation * p<0.05, ** p<0.01, *** p<0.001, versus r = 0.

**Figure 4:**
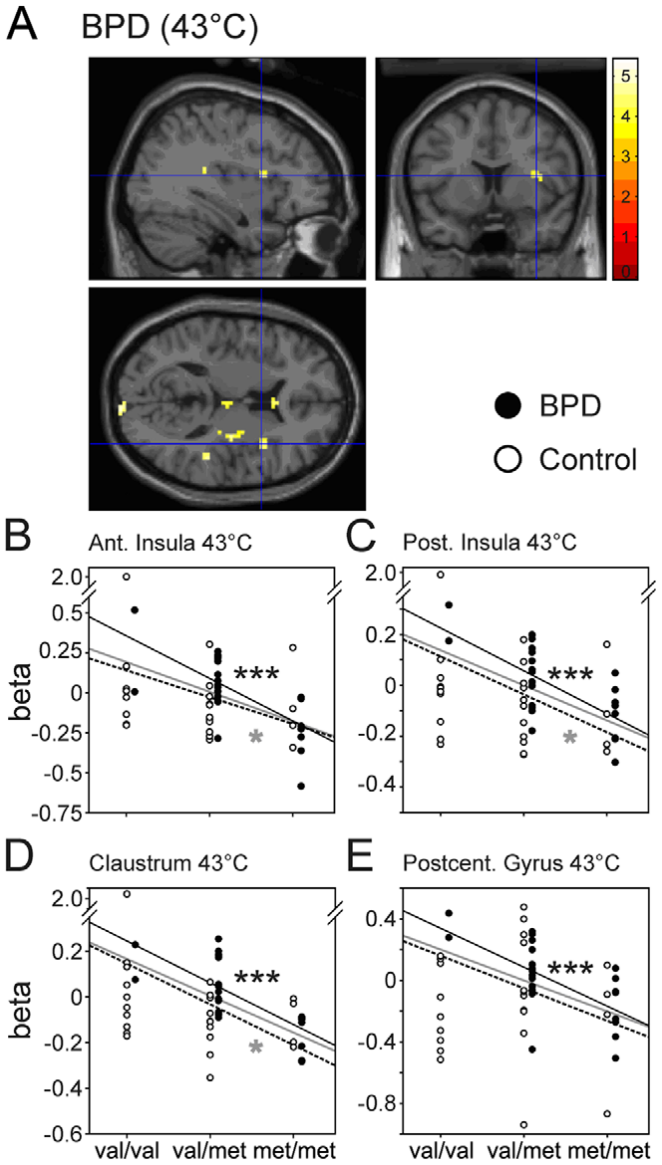
Association between COMT genotype and fMRI activation during painful stimulation in the anterior and posterior insula. Significant correlations were observed during condition 1 within the anterior (A,B) and posterior insula (C), claustrum (D) and postcentral gyrus (D). Labeling as in Fig. 2, * p<0.05, ** p<0.01.

**Figure 5:**
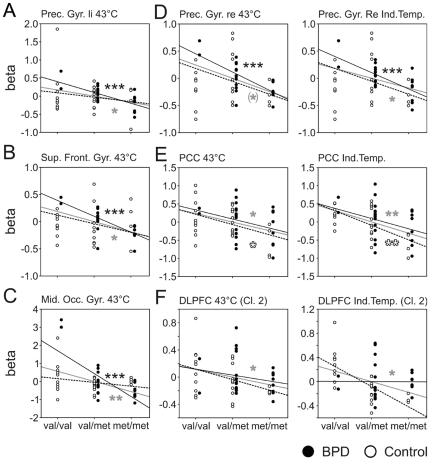
Association between COMT genotype and fMRI activation during painful stimulation in additional brain areas. (A) Left precentral gyrus, (B) Superior frontal gyrus, (C) Middle occipital gyrus, (D) Right precentral gyrus, (E) Posterior cingulated cortex, (F) Dorsolateral prefrontal cortex. Statistics and regression lines for group means are shown in gray (all 50 subjects), black (BPD), and dashed (Control), respectively; significant Pearson product moment correlation * p<0.05, ** p<0.01, *** p<0.001, versus r = 0.

Significant associations between COMT genotype and BOLD signal were observed for both stimulus conditions, in all 50 participants in dorsolateral prefrontal cortex (DLPFC), posterior parietal cortex (PPC), lateral globus pallidus (LGP), and posterior cingulate cortex (PCC). COMT genotype and BOLD signal were significantly correlated in anterior and posterior insula as well as claustrum during *condition 1*, only (Fig. 3, Fig. 4, Fig. 5E,F). In the subgroup of BPD patients, pain processing in *condition 1* was additionally correlated with the number of *val* alleles in postcentral gyrus, left and right precentral gyrus, superior frontal gyrus (BA 6), and middle occipital gyrus (Fig. 4E, Fig. 5A–D, Table 1). Within the subgroup of healthy control participants, pain processing in the higher intense heat stimulation during condition 2 was positively correlated to the number of *val* alleles in DLPFC, PPC, LGP, and PCC (Fig. 3, Fig. 5E, Table 1).

**Table 1:** Group-specific regression analyses.

Healthies					
	Region	Hemisphere	Brodmann Area	z-score	k (cluster level)
**43°C (condition1)**	*No suprathreshold clusters*				
**Individual temperature (condition 2)**	Posterior Parietal Cortex	L	40	4.29	139
	Lateral Globus Pallidus	R	-	3.60	35
	DLPFC	L	46	4.00	33
	DLPFC	L	46	3.45	16
	Posterior Cingulate Cortex	L	23	3.43	14
**BPD**					
**43°C (condition 1)**	Claustrum/Lentiform Nucleus	R	-	3.70	72
	Precentral gyrus	R	-	3.86	26
	Postcentral gyrus	R	-	3.42	26
	Middle occipital gyrus	R	18/19	3.87	21
	Posterior insula	R	-	3.76	19
	Precentral gyrus	L	-	3.70	15
	Anterior insula	R	-	3.69	11
	Superior frontal gyrus	R	6	4.05	11
**Individual temperature (condition 2)**	*No suprathreshold clusters*				

**Figure 6:**
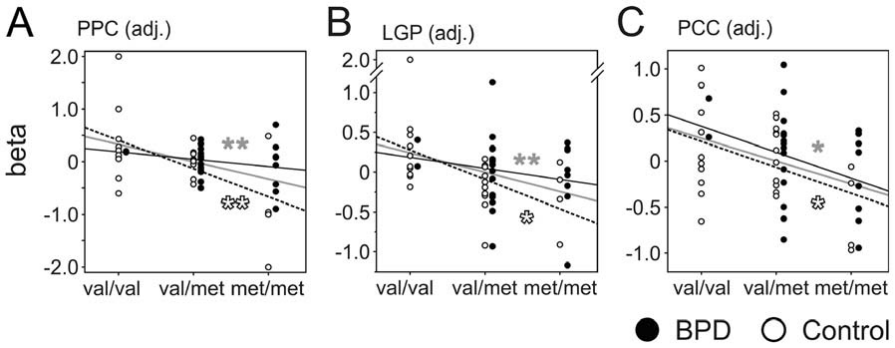
Brain areas activated in response to subjectively adjusted heat pain correlate with COMT val158met polymorphism. During subjectively similar intense heat pain perception (individual temperature in BPD 43°C in healthy controls), posterior parietal cortex (A), lateral globus pallidus (LGP; B) and the posterior cingulate cortex (PPC; C) displayed significant correlation with COMT-polymorphism. Labelling as in Fig. 2,* p<0.05.

As mentioned above, stimulation with 43°C (*condition 1*) induced comparable subjective heat pain sensations in healthy controls as significantly higher heat stimuli applied in BPD in *condition 2* (Fig. 1). Therefore, we directly compared these subjectively adjusted conditions and found a significant correlation of the COMT genotype and fMRI activation in PPC, LGP, and PCC (Fig. 6).

## Discussion

In this study, we used a combined fMRI and molecular genetic approach in the assessment of pain processing. To investigate the association of altered pain sensitivity with the COMT genotype on a more fine-granulated level, we used fMRI correlates of thermal pain stimulation. Here, the number of COMT *val158* alleles was correlated with the activity of several pain-processing brain regions. These included DLPFC, PPC, LGP, PCC, claustrum as well as anterior and posterior insula. In healthy persons, we found the number of *val* alleles to be correlated with cognitive aspects of pain processing in DLPFC and PPC. In patients with borderline personality disorder, a psychiatric condition characterized by reduced pain sensitivity in conjunction with self-injurious behavior, the number of *val* alleles was positively correlated with brain activation in regions of affective pain processing, such as anterior insula. In both groups, basal ganglia activity during painful heat stimulation was also correlated with the number of *val* alleles.

The association of the *val158* allele with activation in DLPFC and, potentially, in PPC, may be related to a specific role of this brain region in pain control [3]. The DLPFC participates in a pain control network, which regulates cortico-subcortical and cortico-cortical loops by inhibiting ascending pathways through the ACC. The study by Lorenz et al. [3] found an inverse correlation between the subjective unpleasantness and the perceived intensity of a heat stimulus and the activation of DLPFC. The interaction of pain control mechanisms and the activity of the endogenous opioid system, which may be influenced by COMT activity, is of particular interest and deserves further research.

Previous investigations revealed a disturbance of affective pain processing in patients with BPD and a related psychiatric condition, posttraumatic stress disorder (PTSD) [21], [22], [27]. In patients with PTSD, elevated pain-related activity in the anterior insula, a brain region related to affective pain processing, could be demonstrated [27]. In the current study, activity in the anterior and posterior insula was related to COMT activity, particularly in borderline patients. The insula plays an important role in monitoring internal bodily states [28], and increased anterior insula activity was found in patients with BPD as well as in patients with PTSD during dissociative states, which are characterized by depersonalisation, derealisation, and reduced pain perception [29], [30].

In both groups, COMT activity as measured by the number of *val* alleles was positively correlated with brain activity in several parts of the basal ganglia. In carfentanil-PET studies [8], [31], a significant influence of the *val158met* polymorphism on the activity of the endogenous opioid system in the striato-pallidal circuits could be shown. Our data of COMT-related activation patterns in these regions support these previous findings and suggest a role of the interaction of the dopamine and the endogenous opioid system.

The association between the activity in DLPFC, PPC and LGP with COMT did not critically depend upon stimulus intensity and/or subjective heat pain sensation. In contrast, PCC displayed significant correlation with COMT polymorphism only at a higher level of subjectively adjusted heat pain perception. This brain region was suggested to be involved in orientation toward noxious somatosensory stimuli and to represent an emotional pre-processor that might assist in establishing the personal relevance of sensory information that comes into the cingulate gyrus [30]. Thus, inactivating PCC - which is active during emotion and non-emotion conditions - may especially reduce perception of noxious stimulation and suffering from pain as suggested by Vogt [30].

Regarding pain sensitivity, we found a correlation with the number of *met* alleles for the induction of a constant pain sensation in healthy subjects (Fig. 2B). Here, the stimulus intensity necessary to induce a painful sensation of NRS 40 decreased with the number of met alleles. This observation resembles the findings of Zubieta [8] who found that the infused volume necessary for the induction of a constant sustained pain perception decreased with the number of *met* alleles. Furthermore we found that the well-known hypoalgesia in borderline personality disorder for thermal pain [20], [22], [26] is not simply explained by the COMT *val158met* polymorphism. The impact of diagnosis as opposed to genotype also becomes apparent when comparing subgroups with the same genotype: The large subgroups of heterozygote patients and controls (middle columns in Fig. 2A) still showed markedly reduced pain sensitivity.

As a limitation of our study it should be mentioned, that we only focussed on one COMT polymorphism. Several SNPs have been described in COMT and three COMT haplotypes comprising the *val158met* polymorphism were found to account for more than 10% of the variance in pain sensitivity in healthy human subjects [9]. However, a recently conducted model comparison based on the Akaike Information Criterion and the Bayesian Information Criterion reveals that the simplest model, comprising only the *val158met* polymorphism, to be the most informative one [32].

In summary, inter-individual differences in neural pain processing in healthy people as well as in patients with a disorder associated with reduced pain sensitivity are significantly influenced by genetic variations in the COMT gene. Differential modulation of brain areas by the COMT polymorphism led to similar painful experiences in our two samples. However, BPD patients and healthy control participants differed regarding those modulatory mechanisms. In healthy participants, the frequency of *val* alleles was associated with higher activity in regions processing cognitive aspects of pain, presumably controlling pain, particularly at higher pain intensities. In contrast, BPD patients revealed an association of COMT activity with affective evaluation of pain mainly at lower stimulus intensities.
